# The effect of protocatechuic acid on nephrotoxicity induced by gentamicin in rats

**DOI:** 10.1007/s00210-025-04064-4

**Published:** 2025-06-25

**Authors:** Handan Mert, Salih Cibuk, Serkan Yildirim, Nihat Mert

**Affiliations:** 1 Department of Biochemistry, Faculty of Veterinary Medicine, Van Yuzuncu Yil University, Van, Turkey; 2 Vocational School of Health Services, , Van Yuzuncu Yil University, Van, Turkey; 3 Department of Pathology, Faculty of Veterinary Medicine, Ataturk University, Erzurum, Turkey; 4 Department of Pathology, Faculty of Veterinary Medicine, Kyrgyz-Turkish Manas University, Chingiz Aitmatov Campus, Djal, Bishkek, 720038 Kyrgyzstan

**Keywords:** Gentamicin, Nephrotoxicity, Protocatechuic acid, Oxidative stress, Antioxidant enzymes

## Abstract

Gentamicin (GM) is an aminoglycoside antibiotic widely used to treat gram-negative infections. Oxidative stress is known to play an important role in the nephrotoxicity of gentamicin. Therefore, the aim of this study was to investigate the possible protective effect of protocatechuic acid (PCA), which is believed to have antioxidant properties, on nephrotoxicity induced by gentamicin. For this purpose, 32 rats were randomly divided into four groups: control (oral physiological saline), PCA (20 mg/kg orally), GM (80 mg/kg/day/i.p.), GM+PCA (80 mg/kg/day/i.p. GM and 20 mg/kg PCA orally). The sampling period was eight days. Blood samples were collected for biochemical analysis and kidney samples for immunohistochemical and histopathological examination. Serum levels of urea, creatinine, Na, K and Cl were measured using an autoanalyzer, while analyses of malondialdehyde (MDA), advanced oxidation protein products (AOPP), gutathione (GSH), superoxide dismutase (SOD), catalase (CAT) and glutathione peroxidase (GPx) were analyzed by ELISA. While the values ​​of urea (p<0.001), creatinine (p<0.001), MDA (p<0.05) and AOPP (p<0.05) decreased in the GM+PCA group compared to the GM group, the values ​​of GSH (p<0.05) and GPx activity (p<0.05) increased. In conclusion, in GM-induced nephrotoxicity, PCA prevented lipid peroxidation and protein oxidation, increased GSH levels and GPx activity, and reduced tubular epithelial necrosis, glomerular atrophy, 8-OHdG and Kim-1 expression in renal cells, according to histopathological and immunohistochemical results. This study once again highlighted that PCA is a good antioxidant, and it can be said that PCA has a protective effect against nephrotoxicity caused by GM.

## Introduction

Gentamicin (GM) is an aminoglycoside antibiotic that is still widely used in the treatment of serious, life-threatening infections caused by gram-negative aerobes. Despite its beneficial effects, low cost, and low levels of resistance, its use is limited due to serious side effects such as ototoxicity and nephrotoxicity, which affects 10–20% of patients (Ali 1995; Cui et al. 2019). Although the mechanism of GM-induced nephrotoxicity is not fully understood, oxidative stress is thought to play a central role (Abdel-Naim et al. 1999; Randjelovic et al. 2017). In vivo and in vitro studies have shown that GM treatment causes oxidative stress (Juan et al. 2007; Karatas et al. 2004; Kandeil et al. 2018a). The current oxidative stress is mediated by H_2_O_2_ and hydroxyl radicals and superoxide anion (Basnakian et al. 2002). GM directly increases ROS production in mitochondria (Morales et al. 2010). The formed ROS inhibit the respiratory chain and ATP production, stimulate the release of cytochrome C and other proapoptotic factors, impair cellular function by damaging cellular proteins, lipids, and nucleic acids, induce mesangial contraction, cause endoplasmic reticulum stress, interfere with inflammation, cell swelling, and necrosis. It inhibits transmembrane sodium transport.

Numerous studies report the protective effect of treatment with substances with antioxidant properties against kidney damage caused by GM (Randjelovic et al. 2012a, b; Stojiljkovic et al. 2012; Kandeil et al. 2018b). This may be due to the protective effect of antioxidants; this is the result of actions that interact at different levels, such as attenuation of the cytotoxic effect of GM, inhibition of vasoconstriction and contraction of the mesangium, anti-inflammatory effect and reduction of lipid peroxidation (Randjelovic et al. 2017).

Protocatechuic acid (PCA, 3,4-dihydroxybenzoic acid), found in many food plants such as olives and white grapes, is an important metabolite of complex polyphenols, particularly anthocyanins (Semaming et al. 2015). PCA is particularly important from a nutritional perspective because it is a major anthocyanin metabolite that can reach tissues in amounts that may exert biological effects on health (Kay et al. 2009). In vivo studies have shown that male balb/cA mice fed a standard diet supplemented with PCA had increased levels of PCA in plasma and tissues such as the brain, heart, liver, and kidney (Lin et al. 2011). It has been reported to possess antioxidant, anti-inflammatory, antibacterial, anticancer, antidiabetic, antiaging, antiulcer, antifibrotic, analgesic, antiviral, antiatherosclerotic, antihyperlipidemic, as well as nephroprotective, neuroprotective, and hepatoprotective activities (Kakkar and Bais 2014; Mert et al. 2024). The antioxidant effect of PCA is 10 times higher than that of α-tocopherol (Lee et al. 2017). Its antioxidant activity is attributed to phenolic hydroxyl groups and activation of endogenous antioxidant enzymes (Han et al. 2018).

In recent years, PCA has attracted the attention of researchers due to its antioxidant activity. However, studies examining the possible effects of PCA on renal protection are very few (Adefegha et al. 2015; Lin et al. 2011; Molehin et al. 2019; Yamabe et al. 2015; Yüksel et al. 2017). Therefore, the aim of this study is to examine the effect of protocatechuic acid on the nephrotoxicity of GM. In particular, the antioxidant property of protocatechuic acid will be investigated with the parameters to be examined.

## Materials and method

### Animals

The animal material was obtained from the Animal Experimental Unit of Van Yuzuncu Yil University. Thirty-two female Wistar albino rats weighing 200–300 g, were used in this study. During the experiment, the rats were housed in rooms with 12 h of darkness/light and a temperature of 22 ± 2 °C, in cages with constant food and fresh water ad libitum. This study was carried out with the approval of the Local Ethics Committee for Animal Experiments of Van Yuzuncu Yil University (27.04.2023, 2023/06–13).

## Experimental application and sample collection

The animals were randomly divided into 4 groups and the experimental period was planned for 8 days.

1-Control group (8 rats): Saline was administered orally for 8 days.

2-Protocatechuic acid group (PCA group) (8 rats): Protocatechuic acid was administered to the animals at a dose of 20 mg/kg orally (Yamabe et al. 2015) for 8 days.

3-Gentamicin group (GM group) (8 rats): Gentamicin was administered at a dose of 80 mg/kg/day/i.p. (Yilmaz et al. 2018) for 8 days.

4-Gentamicin + protocatechuic acid group (GM + PCA group) (8 rats): Gentamicin 80 mg/kg/day/i.p. and protocatechuic acid 20 mg/kg were administered together orally for 8 days.

After the experimental applications (9th day), ketamine 90 mg/kg intraperitoneally was administered to all rats and blood samples were collected. The animals were sacrificed and both kidneys were immediately removed.

### Biochemical analyzes

Blood collected in the tubes was centrifuged at 3000 rpm at + 4 oC for 10 min. The levels of urea, creatinine, Na, K, Cl in the obtained serum samples were performed in an autoanalyzer, and the analyses of MDA, AOPP, GSH, SOD, CAT, GPx were performed in the ELISA device using Sun Red ELISA kits (respectively, Catalog No: 201–11–0157, Catalog No: 201–11–2673, Catalog No: 201–11–7122, Catalog No: 201–11–0169, Catalog No: 201–11–5106, Catalog No: 201–11–5104).

### Histopathological examination

Tissue samples collected at the end of the evaluation were fixed in 10% formaldehyde solution for 48 h and embedded in paraffin blocks at the end of routine tissue follow-up procedures. Sections of 4 μm thickness were taken from each block and the preparations prepared for histopathological examination were stained with hematoxylin–eosin (HE) and examined under a light microscope (Olympus BX 51, Japan). The sections were graded as absent (-), mild ( +), moderate (+ +), and severe (+ + +) according to their histopathological features***.***

### Immunohistochemical examination

Tissue sections taken on adhesive slides (poly-L-lysine) for immunoperoxidase examination were deparaffinized and dehydrated. Then, 10 min in 3% H_2_O_2_, endogenous peroxidase was inactivated. Then, the tissues were boiled in 1% antigen retrieval solution (citrate buffer (pH + 6.1) 100X) and allowed to cool to room temperature. In order to avoid nonspecific background staining in the tissues, the sections were washed with protein block for 5 min and allowed to incubate. Then, the primary antibody (8-OHdG and Kim-1; Cat No: sc-66036, sc-518008, dilution rate: 1/100. USA) was dripped onto the tissues and incubated according to the instructions for use. The chromogen 3–3 Diaminobenzidine (DAB) was used as a chromogen in tissues. Stained sections were examined under a light microscope (Zeiss Axio, Germany).

### Statistical analysis

The software package "SPSS Statistic 20" was used for the analysis of biochemical data. Descriptive statistics of the presented characteristics are expressed as mean and standard deviation. One-way ANOVA analysis was performed in the statistical analysis of all parameters. Tukey’s test was used to compare different groups. The statistical significance level was taken at 5% in the calculations.

The Kruskal–Wallis non-parametric test was used for the analysis of differences between groups in semi-quantitative data obtained during histopathological examination, and the Mann Whitney U test was used for the comparison of paired groups. The software package SPSS 13.0 was used for these statistical analyses.

In order to determine the intensity of positive staining from the pictures obtained as a result of immunohistochemical staining; 5 random areas were selected from each image and evaluated in the ZEISS Zen Imaging Software program. Data were statistically defined as mean and standard deviation (mean ± SD) for % area. One-way ANOVA followed by Tukey’s test was performed to compare positive immunoreactive cells and immunopositive stained areas with healthy controls. As a result of the test, a value of *p* < 0.05 was considered significant and the data were presented as mean ± SD.

## Results

### Results of biochemical parameters

The mean serum levels of urea, creatinine, Na, K, Cl of control rats, PCA, GM and GM + PCA groups are shown in Table 1.

**Table 1:** Mean values ​​of serum levels of urea, creatinine, Na, K and Cl of control rats, PCA, GM and GM + PCA groups

	n	Control X ± SD	PCA X ± SD	GM X ± SD	GM + PCA X ± SD	*p*
Urea (mg/dl)	8	42.37 ± 9.43^a^	38.07 ± 7.71^a^	158.11 ± 34.12^c^	74.28 ± 14.21^b^	*p* < 0.001
Creatinine (mg/ dl)	8	0.42 ± 0.04^a^	0.36 ± 0.04^a^	2.67 ± 0.40^c^	0.63 ± 0.20^b^	*p* < 0.001
Na (mmol/L)	8	143.2 ± 2.0^a^	141.3 ± 3.2^a^	146.5 ± 5.2^a^	144.7 ± 3.8^a^	*p* > 0.05
K (mmol/L)	8	5.9 ± 0.6^a^	5.8 ± 0.2^a^	5.0 ± 0.6^a^	5.4 ± 0.9^a^	*p* > 0.05
Cl (mmol/L)	8	102.2 ± 1.9^a^	99.0 ± 1.9^a^	98.5 ± 6.9^a^	101.2 ± 6.9^a^	*p* > 0.05

a, b, c: The difference between the means of groups with different letters on the same line is statistically significant

Serum urea and creatinine levels were highest in the GM group but significantly decreased in the GM + PCA group (*p* < 0.001). In addition, statistical significance was not found between the groups in terms of serum Na, K and Cl (*p* > 0.05).

The mean serum MDA, AOPP, GSH levels and SOD, CAT, GPx activities of the rats in the control, PCA, GM, GM + PCA groups are given in Table 2.

**Table 2:** Oxidative stress parameters and antioxidant enzyme activities of rats in control, PCA, GM and GM + PCA groups

	Control X ± SD		PCA Group X ± SD		GM Group X ± SD		GM + PCA Group X ± SD		
	n	X ± Sx	n	X ± Sx	n	X ± Sx	n	X ± Sx	*P*
MDA(nmol/ml)	8	1.27 ± 0.35^ab^	8	1.2 ± 0.23^a^	8	1.96 ± 0.81^b^	8	0.76 ± 0.4^a^	*p* < 0.05
AOPP(nmol/ml)	8	12.2 ± 2.59^ab^	8	12.0 ± 1.73^ab^	8	15.46 ± 4.51^b^	8	11.48 ± 1.73^a^	*p* < 0.05
GSH (mg/L)	8	260.91 ± 53.26^b^	8	244.24 ± 57.45^b^	8	101.85 ± 16.57^a^	8	215.85 ± 53.15^b^	*p* < 0.05
SOD (ng/ml)	8	9.5 ± 4.23^b^	8	7.77 ± 1.56^ab^	8	4.61 ± 0.74^a^	8	6.19 ± 1.29^a^	*p* < 0.05
CAT (ng/ml)	8	19.71 ± 5.68^a^	8	20.58 ± 5.42^a^	8	15.3 ± 4.74^a^	8	18.65 ± 5.76^a^	*p* > 0.05
GPx (ng/ml)	8	76.52 ± 0.9^b^	8	70.54 ± 8.38^ab^	8	58.89 ± 2.64^a^	8	71.55 ± 14.98^b^	*p* < 0.05

a, b:. The difference between the means of groups with different letters on the same line is statistically significant

While MDA (*p* < 0.05) and AOPP (*p* < 0.05) levels decreased in GM + PCA group compared to GM group, GSH level (*p* < 0.05) and GPx activity (*p* < 0.05) were observed to statistically increase. Again, there was no statistical significance between these two groups in terms of SOD and CAT activities (*p* > 0.05).

### Histopathological results

#### Control group

**Fig. 1:**
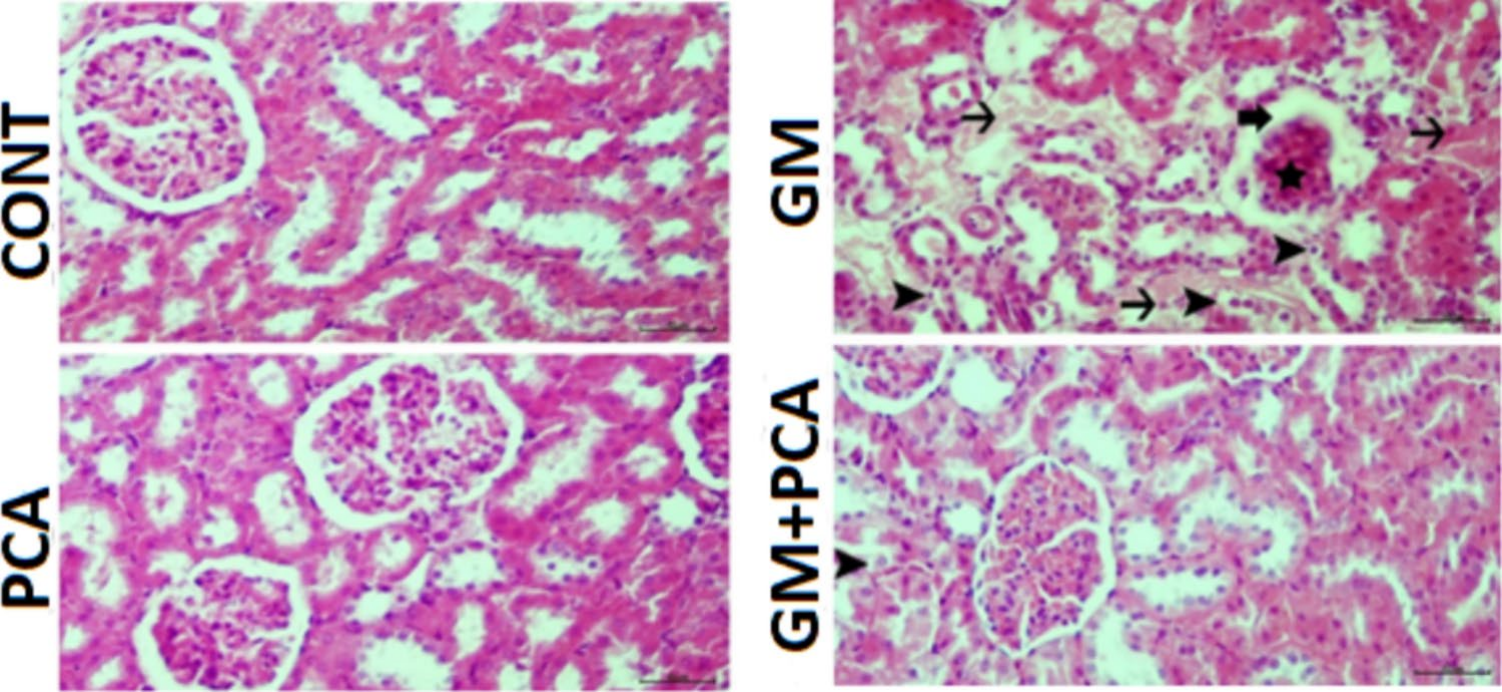
Kidney tissue, degeneration of tubular epithelium (arrowheads), necrosis (thin arrows) atrophy of the glomerulus (star), dilatation of Bowman’s capsule (thick arrow). H&E

Upon histopathological examination of renal tissues, it was observed that they had a normal histological structure (Fig. 1).

#### PCA group

Upon histopathological examination of renal tissues, it was determined that they had a normal histological appearance (Fig. 1).

#### GM group

Upon histopathological examination of renal tissues, severe hydropic degeneration and necrosis of the renal tubular epithelium, moderate atrophy of the glomerulus, moderate dilatation of Bowman’s capsule, and severe hyperemia of the vessels were observed (Fig 1).

#### GM+PCA group

Upon histopathological examination of renal tissues, mild degeneration of the tubular epithelium, mild dilatation of Bowman’s capsule, and hyperemia of the vessels were observed (Fig 1). A statistically significant difference (p˂0.05) was found compared with the GM group. Histopathological results are summarized in Table 3.

**Table 3:** Scoring of histopathological findings in kidney tissues and statistical results

	Control	PCA	GM	GM + PCA
Degeneration of tubular epithelium	-	-	+ + +	+
Necrosis of tubular epithelium	-	-	+ + +	-
Atrophy of the glomeruli	-	-	+ +	-
Dilatation of Bowman’s capsule	-	-	+ +	+
Hyperemia in the veins	-	-	+ + +	+ +

### Immunohistochemical Results

When renal tissues were examined by immunohistochemistry;

#### Control group

**Fig. 2:**
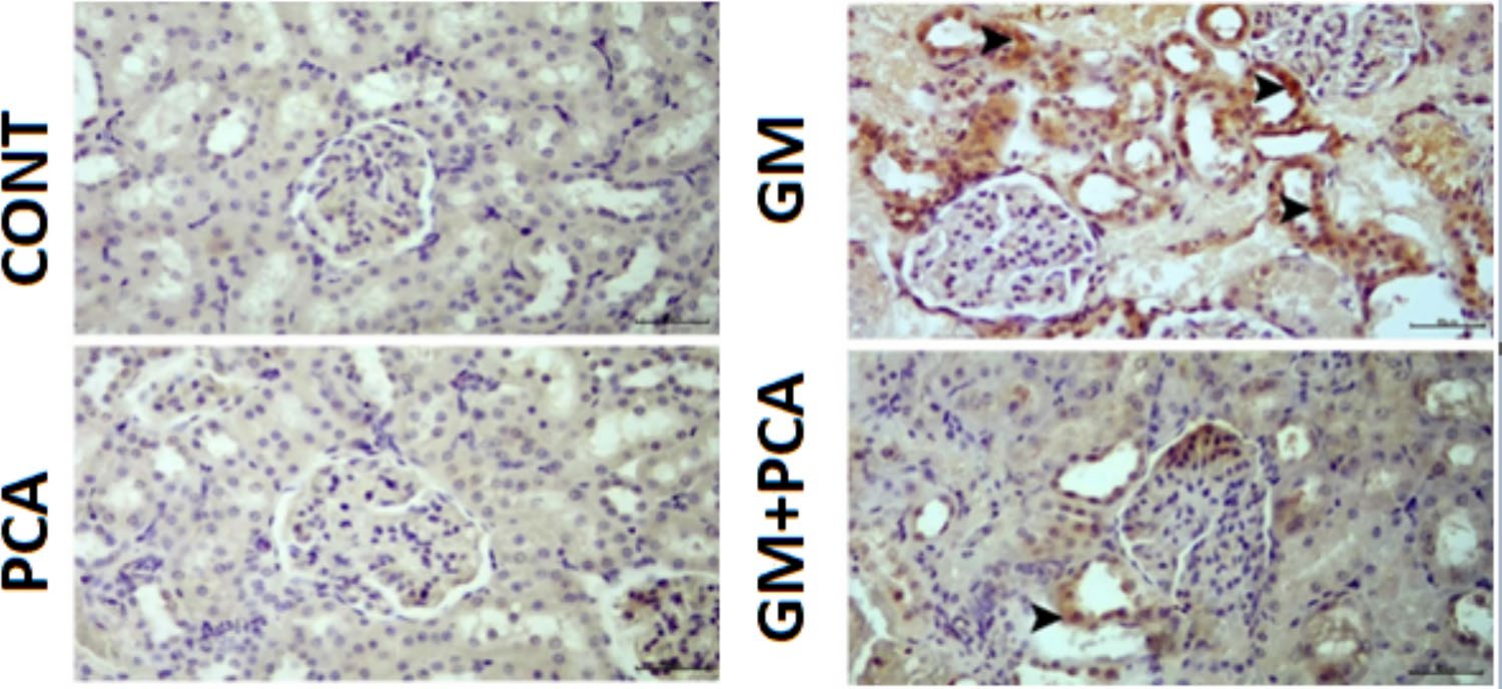
Cytoplasmic 8-OHdG expression (arrowheads) in tubular epithelia. IHC-P, Bar: 40µm

**Fig. 3:**
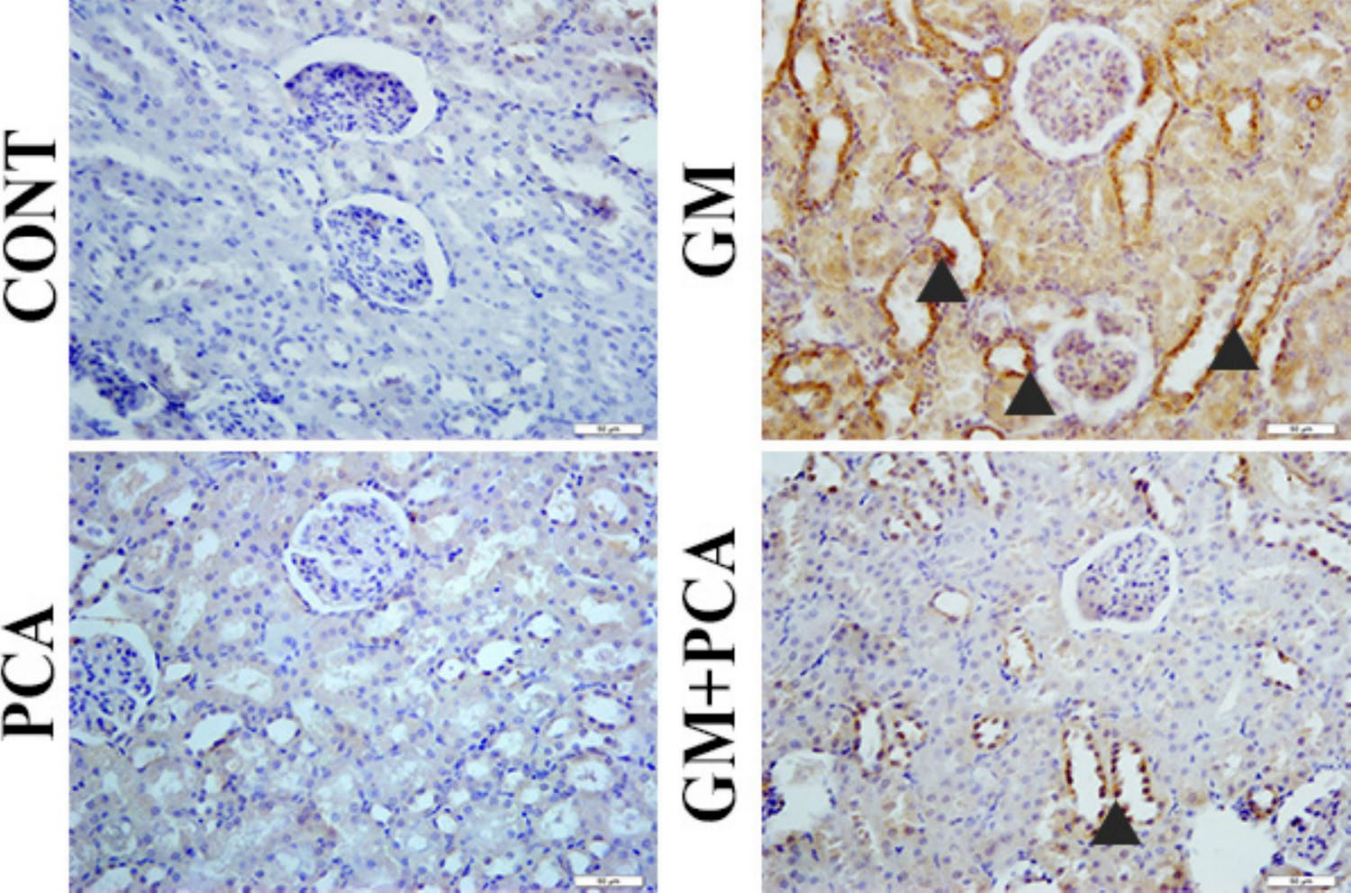
Cytoplasmic Kim-1 expression in tubular epithelia (arrowheads), IHC-P, Bar: 40µm

8-OHdG and Kim-1 expressions were negatively evaluated (Figs. 2, 3).

#### PCA group

8-OHdG and Kim-1 expressions were negatively evaluated (Figs. 2, 3).

#### GM group

Severe levels of 8-OHdG and Kim-1 expressions were observed in the cytoplasm of tubular epithelium (Figs. 2, 3).

#### GM+PCA group

Slight levels expressions of 8-OHdG and Kim-1 expressions were observed in the tubular epithelium (Figs. 2, 3).

**Fig. 4:**
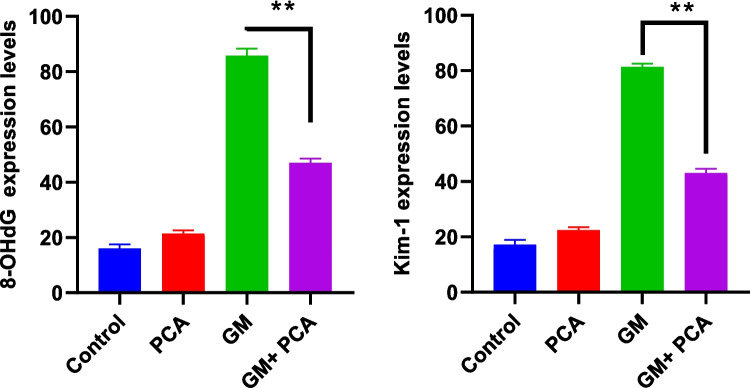
Statistical data of immunohistochemical findings observed in kidney tissues. 8 OHdG and Kim-1 expressions, (** p=0.0022)

A statistically significant differencein the GM+PCA group was found compared with the GM group. The immunohistochemical results are summarized in Fig. 4.

## Discussion and conclusion

The kidney is an essential organ that performs many important functions including maintaining homeostasis, regulating the extracellular environment such as detoxification, and excreting toxic metabolites and drugs (Ferguson et al. 2008; Kim and Moon 2012). Therefore, the kidney can be considered as the major target organ for exogenous toxic substances. It not only has a rich blood supply that takes 25% of the cardiac output, but also helps in eliminating these toxins through glomerular filtration and tubular secretion (Patel Manali et al. 2011). The kidneys are prone to drug-induced injury due to this high relative blood flow (Eman et al. 2020). Gentamicin, an aminoglycoside antibiotic, is one of the major causes of drug-induced nephrotoxicity.

Nephrotoxicity is a specific feature of the kidneys in which excretion is not smooth due to toxic chemicals or drugs (Finn and Porter 2003; Galley 2000; Kim and Moon 2012). Serum urea and creatinine levels are well-established biomarkers for nephrotoxicity and renal dysfunction (Al-Naimi et al. 2019). In many studies, creatinine and urea levels were found to be increased in animals treated with GM (Ataman et al. 2018; Yilmaz et al. 2018; Botros et al. 2022; Erseckin et al. 2022; Sarwar et al. 2022). Adefegha et al. (2015) suggested that the elevated levels of urea, uric acid, and creatinine, which were in cadmium-induced nephrotoxicity, were also decreased due to the renal protective effect of PCA. In this study, it was determined that serum urea and creatinine levels increased in GM group animals and decreased with PCA administration (*p* < 0.001), indicating an improvement in renal functions. Indeed, during histopathological examination of renal tissues in the GM group, severe hydropic degeneration and necrosis of the renal tubular epithelium and moderate atrophy of the glomeruli were observed, while in the GM + PCA group, no necrosis of the tubular epithelium and no atrophy of the glomeruli were observed. In addition, according to immunohistochemical results, the expression of 8-OHdG and Kim-1 in renal cells decreased with PCA administration.

The kidneys play a very important role in regulating the body’s water and electrolyte balance. Therefore, in case of renal failure, water, electrolyte and acid–base balance disorders may occur. Kidney diseases are often associated with hypervolemia, hyperkalemia, hypocalcemia, hyperphosphatemia, hyponatremia, hypermagnesemia and metabolic acidosis. The severity of these electrolyte disorders reflects the patient’s catabolic state and the degree of kidney damage (Caliskan and Yildiz 2010; Medineli et al. 2021).

Changes in urinary excretion of some ions are observed in renal damages after gentamicin treatment. Decreased Na, K-ATPase activity in proximal tubules in rats given GM may be due to GM-induced nephrotoxicity. Indeed, this enzyme is responsible for regulating intracellular electrolyte transport and cell volume (Ali 1995). Aminoglycoside nephrotoxicity has been reported to cause decreased serum potassium levels in experimental animals and humans (Cronin and Thompson 1991; Silan et al. 2007). In addition, GM-induced glomerular dysfunction has been associated with increased plasma sodium levels (Cuzzocrea et al. 2002). Medineli et al. (2021) found that Na and K levels did not change and Cl levels increased in the GM treated group of rats compared to controls. Noorani et al. (2011) also found that changes in serum Na levels in rats with gentamicin-induced nephrotoxicity were insignificant compared to controls, and potassium and chloride levels were higher than in the control group (*p* < 0.05). Yilmaz et al. (2018) on the other hand found that Na and K levels increased in the GM group compared to controls, but this was not significant, and Cl levels did not change. In this study, serum Na, K, and Cl levels were examined. There was no statistical significance between groups in terms of serum Na, K, and Cl levels. Despite changes in renal electrolyte distribution with GM administration to animals, aminoglycoside-induced changes in plasma electrolytes were occasionally observed. This may be due to the presence of excess electrolytes in standard laboratory animal diets or the absence of other factors predisposing to electrolyte imbalance (Bach and Lock 2012).

The details of the mechanisms of GM-induced nephrotoxicity are not yet fully understood. Various mechanisms such as oxidative stress, apoptosis, tubular necrosis, phospholipidosis, increased endothelin I, and leukocyte infiltration have been suggested by different studies (Ahmadvand et al. 2020; Balakumar et al. 2008; Lopez-Novoa et al. 2011). GM are taken up by renal tubular cells via the anion transport system. Accumulation of GM in these cells ultimately leads to morphological changes, functional alterations, and increased reactive oxygen species (ROS) and reactive nitrogen species (RNS) in the kidney (Ahmadvand et al. 2020; Balakumar et al. 2008). In case of nephrotoxicity, these free radicals promote the inflammatory process, apoptosis, and necrosis (Ahmadvand et al. 2020; Lopez-Novoa et al. 2011). Free radicals also suppress the renal antioxidant system through protein oxidation (Sener et al. 2002) and lipid peroxidation (LPO) (Nitha and Janardhanan 2008).

Since oxidative stress plays a role in the pathogenesis of chronic inflammatory diseases, modulating the cellular redox state by enhancing endogenous antioxidant defenses may be an effective mechanism in disease prevention. In this context, dietary polyphenols generally act as antioxidant compounds, although to varying degrees. Polyphenols exert indirect antioxidant effects through the induction of genes involved in the endogenous defense system (Masella et al. 2004; Varì et al. 2011). The endogenous defense system, composed of enzymatic antioxidants such as superoxide dismutase, catalase, glutathione reductase, and glutathione peroxidase, and non-enzymatic antioxidants such as GSH, plays an important role in protecting cells from oxidative damage caused by electrophiles and reactive oxidants (Varì et al. 2011). PCA has shown a particularly high ability to induce gene expression of antioxidant enzymes. In a study using the J774 A.1 macrophage cell line, PCA was found to increase the expression of GPx and GR, mainly by inducing JNK-mediated phosphorylation of the transcription factor Nrf2, which is the main regulator of antioxidant/detoxification (Varì et al. 2011). Again, PCA has been shown to mediate hepatoprotection by increasing SOD, CAT, GST, and NQO-1 activities via Akt and PI3K (Ibitoye and Ajiboye 2020).

The antioxidant potential of PCA has been shown to be ten times higher than that of α-tocopherol (Song et al. 2020). PCA donates the hydroxyl groups of its chemical structure as a hydrogen atom donor for the reduction of peroxyl radicals, stopping their harmful effects on the cell membrane and cellular components (Owumi et al. 2019). PCA; in addition to stimulating the activities of endogenous antioxidant enzymes such as CAT, SOD, GST, GR, and GPx, they also reduce the levels of ROS and MDA (Li et al. 2021; Song et al. 2020). In PCA-mediated hepatorenal protection, GSH level increases significantly and thus increases the bioavailability of cellular GSH to scavenge the produced free radicals (Kassab et al. 2022; Owumi et al. 2019).

Masella et al. (Masella et al. 2004) found that biophenols from extra virgin olive oil, namely PCA and oleuropein, completely prevent J774 A.1-mediated LDL oxidation, inhibit O2•- and H_2_O_2_ production and decrease GSH content, thereby counteracting time-dependent changes in intracellular redox balance, re-regulate GR and GPx activities, restoring γGCS, GR and GPx mRNA expression to control values. They reported that activation of mRNA transcription of GSH-related enzymes represents an important mechanism in the phenolic antioxidant effect. Again, recent studies strongly suggest that dietary polyphenols can stimulate antioxidant transcription and detoxification defense systems through antioxidant responsive elements (AREs) (Masella et al. 2005).

In this study, MDA and AOPP levels were examined as parameters of oxidative stress. It was determined that MDA and AOPP levels, which increased in the GM group, decreased significantly with PCA administration. The fact that the values ​​obtained were even lower than those in the control group can be interpreted as the fact that PCA behaves as an antioxidant compound due to its chemical structure. Indeed, the antioxidant capacity of a phenolic compound depends on some factors such as the structure of the phenolic compound, the number of aromatic and hydroxyl groups in its structure, and the distribution of these groups within the structure (Adefegha et al. 2015; Balasundram et al. 2006). Again in this study, the reduced level of GSH and the activity of the antioxidant enzyme GPx in the GM group increased significantly with PCA administration (*p* < 0.05). PCA can be said to increase GSH bioavailability (Kassab et al. 2022; Owumi et al. 2019) and induce the synthesis of antioxidant enzymes by increasing GPx activity. Indeed, modulatory effects of PCA on GSH-related enzymes have been identified in previous in vitro studies (Masella et al. 2004).

In conclusion, in this study, the protective effect of PCA on GM-induced nephrotoxicity was investigated. In GM-induced nephrotoxicity, PCA prevented lipid peroxidation and protein oxidation, increased GSH level and GPx activity, and according to histopathological and immunohistochemical results, it prevented tubular epithelium necrosis, glomerular atrophy, decreased 8-OHdG and Kim1-expression. With this study, it was emphasized once again that PCA is a good antioxidant agent and it can be said that PCA has a protective effect in GM-induced nephrotoxicity.

## Data Availability

All source data for this work (or generated in this study) are available upon reasonable request.
